# Large language models for pneumonia detection in radiology reports via text analysis

**DOI:** 10.1371/journal.pdig.0001104

**Published:** 2026-09-23

**Authors:** Weisheng Chen, Xinya Li, Zhongkai Qu, Ting Liu, Bo Chen, Liying Huang, Ningxia He, Zhigang Wang, Jun Lyu

**Affiliations:** 1 Department of Intensive Care Unit, The First Affiliated Hospital of Jinan University, Guangzhou, China; 2 School of Nursing, Jinan University, Guangzhou, China; 3 Department of Critical Care Medicine, Shenzhen People’s Hospital, The Second Clinical Medical College, Jinan University, Shenzhen, China; 4 Department of Pharmacy, The First Affiliated Hospital of Jinan University, Guangzhou, China; 5 Clinical Research Center, The First Affiliated Hospital of Jinan University, Guangzhou, China; Atilim University: Atilim Universitesi, TÜRKIYE

## Abstract

Pneumonia is a common infection in critically ill patients with poor outcomes, and its identification relies heavily on radiological evidence. Most MIMIC-based pneumonia studies rely on structured codes rather than radiological text, which may limit the precision of pneumonia-related case identification. This study evaluated large language models (LLMs) and retrieval-augmented generation (RAG) frameworks for automated detection of radiographic evidence of pneumonia from radiology reports. Radiological reports from the MIMIC-IV database were analyzed using pre-trained models in an inference-only setting without additional training, fine-tuning, or dataset splitting. Model performance was evaluated using accuracy, precision, sensitivity, specificity, and F1-score, with point estimates calculated from the full dataset and corresponding 95% confidence intervals estimated using 1,000 patient-level clustered bootstrap replicates. Independent evaluation was subsequently performed using a GZ dataset from a tertiary hospital in Guangzhou. RAG generally improved model performance, with gemma3-27b achieving the highest accuracy of 96.4% on the MIMIC-IV dataset and maintaining an accuracy of 87.6% on the GZ dataset. Model performance did not increase linearly with parameter size, suggesting that task-specific model suitability may be more important than parameter scale for this application. Although larger models required longer inference times, their increased computational costs did not consistently translate into proportional performance gains. Overall, LLMs demonstrated high accuracy in interpreting radiology reports for detecting radiographic evidence of pneumonia. The RAG architecture further enhanced robustness and generalizability through knowledge augmentation, providing a methodological basis for automated radiology report analysis and large-scale database curation.

## Author summary

In this study, we explored whether modern artificial intelligence tools can help identify radiographic evidence of pneumonia more accurately by reading radiology reports. This condition is a frequent and serious infection in intensive care units, and its diagnosis often depends on how clinicians interpret imaging findings. Many previous studies have relied on coded data, which may miss important details contained in free-text reports. We used large language models and a retrieval-based approach to analyze radiology reports and determine whether pneumonia was present. We found that these models can achieve high accuracy and remain reliable when applied to data from a different hospital, suggesting that the approach is broadly applicable. Importantly, we observed that simply using larger and more computationally expensive models does not always lead to better results. Instead, models that are better aligned with the specific clinical task tend to perform best. Our findings suggest that artificial intelligence can support more precise and scalable identification of radiographic evidence of pneumonia from routine clinical data. This may help improve research quality and support future clinical decision-support applications.

## Introduction

Pneumonia is a prevalent and severe infectious disease in the intensive care unit (ICU). It is categorized as either community-acquired pneumonia (CAP) or hospital-acquired pneumonia (HAP), depending on the place of onset [1]. CAP refers to pulmonary infections that occur prior to hospital admission or within 48 hours of admission, whereas HAP specifically denotes infections developing after 48 hours of hospitalization. The latter is confined to the hospital environment and is often associated with infections caused by drug-resistant pathogens [2]. Patients in the ICU are particularly vulnerable to pneumonia and its severe complications, such as respiratory failure, sepsis, and multiple organ dysfunction. This is due to critical underlying illnesses, frequent invasive interventions, and impaired immune function [3–5]. These factors underscore the critical clinical importance of timely identification of pneumonia-related radiographic findings and precise management. A plethora of epidemiological evidence has been published on the subject, and it is now well established that pneumonia occurs frequently among critically ill patients. The evidence is also strongly suggestive of a link between pneumonia and prolonged hospital stays, increased healthcare costs, and higher mortality rates [6–8].

Although pneumonia is a high-risk condition and a major clinical concern in the ICU, current large-scale critical care databases have notable limitations for research purposes. The majority of extant databases are contingent on clinical diagnoses or International Classification of Diseases (ICD) codes, which frequently exhibit a paucity of precise information regarding the onset or timing of diagnosis and are often devoid of accompanying radiological reports [9–11]. Consequently, this restricts the ability to conduct temporal analyses and to infer causal relationships in studies of pneumonia. In particular, for pneumonia research, the lack of clear temporal markers directly hinders the evaluation of disease onset and the timing of intervention, thus compromising the precision of database-driven epidemiological and prognostic analyses. Therefore, rather than focusing on etiological classification, identifying radiographic evidence of pneumonia from radiological text reports represents a more feasible and objective approach in large-scale database studies. Consequently, the accurate determination of the timing of radiographic evidence of pneumonia through radiological text reports remains a critical and pressing challenge in contemporary ICU pneumonia research.

Imaging examinations are considered the most important component of a pneumonia diagnosis [12]. Radiographic findings on chest X-rays or CT scans not only confirm the presence of pneumonia but also assist in determining the timing of pneumonia onset. In recent years, significant progressions have been witnessed in natural language processing (NLP) and LLMs for the analysis of medical texts [13,14], thereby yielding novel prospects for the extraction of temporal and radiographic data from radiological reports. The utilization of LLMs in this context aims to extract and detect radiographic evidence of pneumonia from textual reports, rather than to establish a clinical diagnosis. However, extant studies have yet to fully harness this technology for systematic detection of radiographic evidence of pneumonia in ICU settings, thereby limiting the utility and research potential of large-scale clinical databases in this domain.

The objective of this study is to apply LLMs to analyze ICU radiology reports and detect radiographic evidence of pneumonia, with a view to addressing the limitations of structured-code-based approaches in existing databases. This study focuses on evaluating the feasibility of automated radiographic evidence detection for retrospective database curation and research case labeling, rather than real-time clinical decision support. The integration of radiological text interpretation with temporal annotation provides a methodological framework that may inform future studies, while emphasizing semantically grounded and reproducible automated identification of pneumonia-related findings.

## Methods

### Ethics statement

This study was conducted using data from the publicly available Medical Information Mart for Intensive Care IV (MIMIC-IV) database and clinical data from the First Affiliated Hospital of Jinan University. The MIMIC-IV database was approved by the Institutional Review Boards of Beth Israel Deaconess Medical Center (Boston, MA, USA) and the Massachusetts Institute of Technology (Cambridge, MA, USA), and all data were de-identified. Consequently, the requirement for informed consent was waived.

The use of clinical data from the First Affiliated Hospital of Jinan University was approved by the Institutional Review Board of the First Affiliated Hospital of Jinan University (Approval No. KY-2025–266). Due to the retrospective nature of the study and the use of anonymized data, the requirement for informed consent was waived.

### Data source and study population

The data sources for this study comprise the MIMIC-IV (version 2.2) database and radiology text reports from a tertiary hospital in Guangzhou. The MIMIC-IV database, a collaborative effort between the Massachusetts Institute of Technology and Beth Israel Deaconess Medical Center, comprises extensive clinical data from patients admitted to ICU between 2008 and 2019 [15–17]. The contents of the EHR system include patients’ demographic information, vital signs, laboratory results, medication and procedure records, clinical notes, imaging reports, and discharge outcomes [18,19].

**Fig 1:**
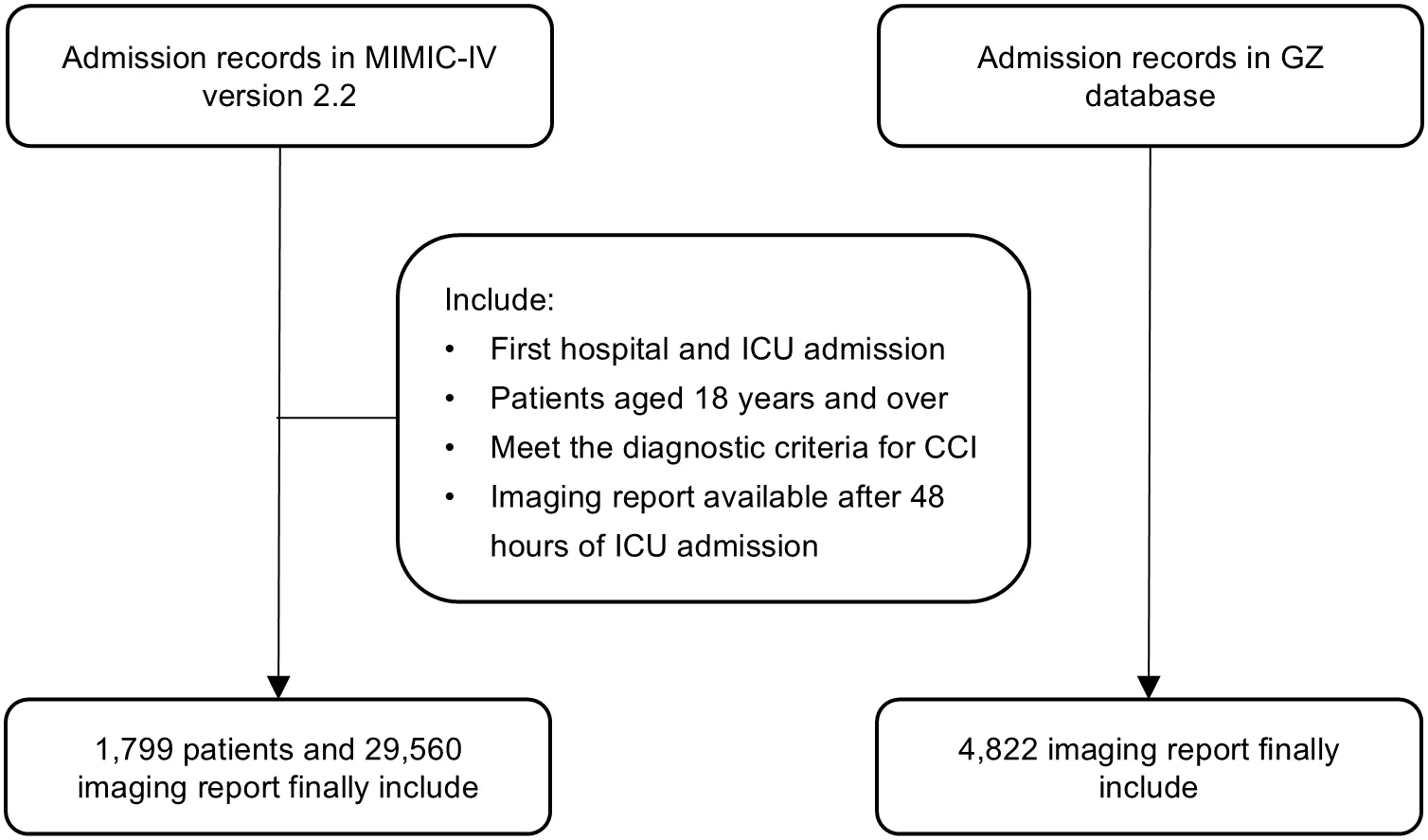
Flowchart of study inclusion criteria. Abbreviations: CCI, chronic critical illness; ICU, intensive care unit; MIMIC IV, medical information mart for intensive care IV.

The MIMIC-IV cohort consisted of patients with CCI. To be eligible, patients were required to be undergoing their first hospitalization and first ICU admission, be aged ≥18 years, and meet the definition of CCI, which is defined as an ICU stay exceeding 14 days [20] (Fig 1). In this study, imaging examinations performed ≥48 hours after ICU admission were used to ensure temporal relevance to in-hospital clinical status. However, this criterion was not intended to distinguish HAP from CAP. The imaging modalities encompassed both chest X-rays (CXR) and chest computed tomography (CT), with the text reports from these investigations serving as the input for model inference and the subsequent performance evaluation. In order to assess the generalizability of the model further, imaging text reports were collected from a tertiary hospital in Guangzhou (GZ Dataset). This dataset consisted of adult ICU patients admitted between 2021 and 2024, without restriction as regards CCI status. The corresponding imaging reports were collected and used for independent external validation of the proposed framework.

### Reference standard

The reference standards employed in this study were established by a multidisciplinary expert panel composed of ICU clinicians, medical doctor candidates with medical imaging backgrounds, and doctoral candidates specializing in critical care medicine research. The panel members had relevant clinical and research experience in interpreting radiology reports. All imaging reports were manually annotated according to standardized criteria in order to guarantee scientific rigor and reproducibility of radiographic evidence identification. The annotation process was conducted in two stages. In the initial round, each expert reviewed reports that had been randomly assigned to them in a single-blind manner. The presence of radiographic evidence of pneumonia was then determined by the experts based on predefined criteria, without any communication between the experts taking place. In the second round, report sequences were re-randomized and cross-assigned to ensure that no expert re-evaluated reports they had previously reviewed. In the event of any discrepancies between rounds, a third-round consensus process was conducted, in which experts from different disciplines jointly reviewed the reports and reached a final decision through discussion. This process allowed integration of complementary domain expertise from intensive care and medical imaging to improve annotation reliability. This multidisciplinary consensus approach was used to integrate complementary clinical perspectives and reduce individual bias. As the final labels were determined through a consensus-based adjudication process rather than independent parallel ratings, inter-rater agreement metrics such as Cohen’s kappa were not calculated. The final labels indicating the presence or absence of radiographic evidence of pneumonia were utilized as the reference standard for this study, thereby providing a robust and reliable foundation for subsequent model performance assessments.

### Models and architectures

**Fig 2:**
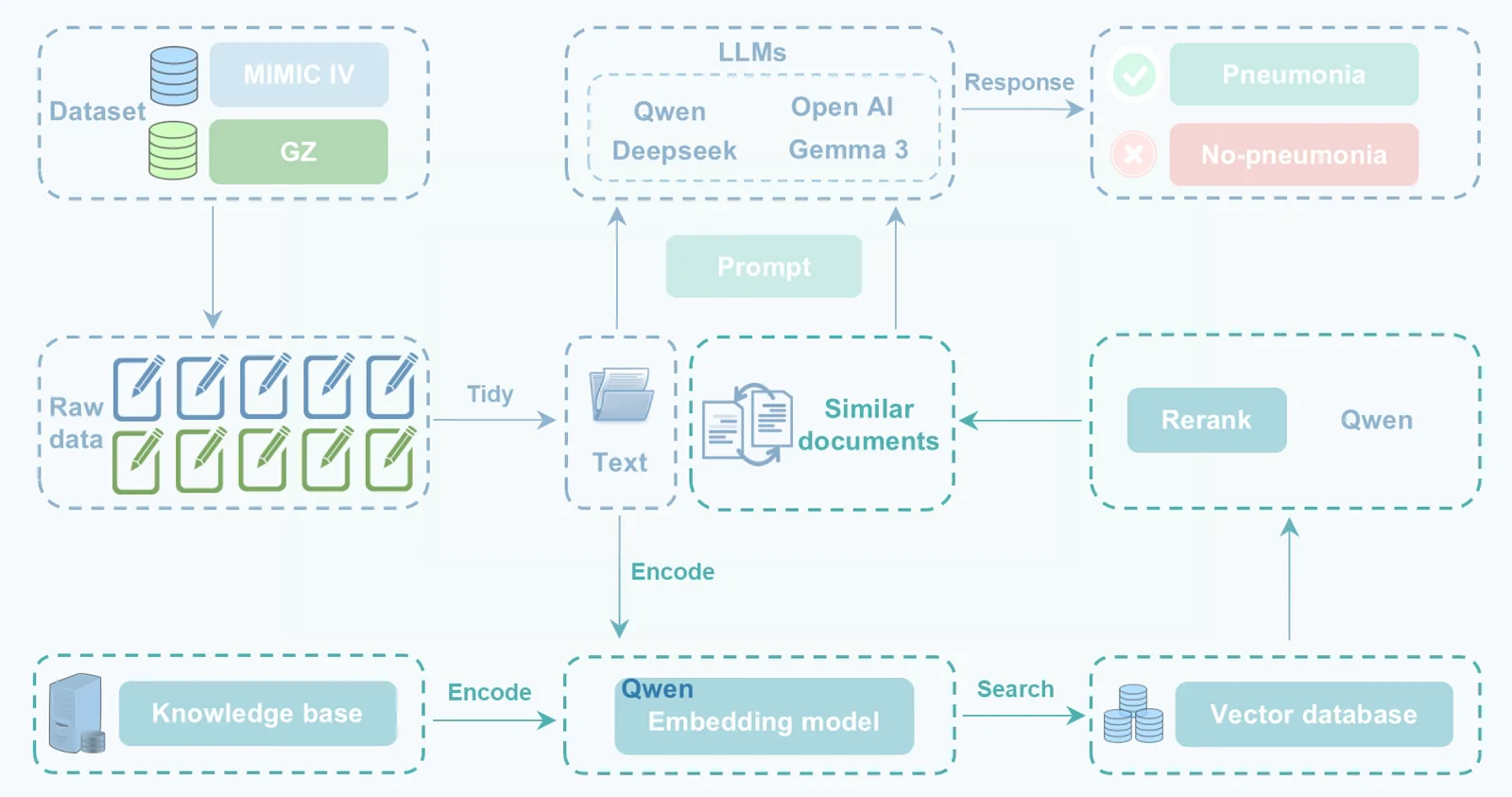
Workflow of LLMs and RAG-enhanced models. Abbreviations: LLM, large language model; MIMIC IV, medical information mart for intensive care IV; RAG, retrieval-augmented generation.

The present study adopts a strategy that integrates LLMs with a retrieval-augmented generation (RAG) framework to achieve automated pneumonia diagnosis from radiology text reports (Fig 2). The LLMs employed in this study include deepseek-r1-0528, the qwen3 series (235b and 32b), gemma3-27b, and gpt-oss-20b [21,22]. Each of these LLMs demonstrates distinct strengths in language understanding, reasoning, and natural language generation (Table 1). By leveraging the complementary capabilities of multiple models, this approach enables a more comprehensive semantic interpretation of complex medical language within radiology reports, thereby improving diagnostic precision.

**Table 1:** Characteristics of the language models included in this study.

Model types	Models	Size	Layers	Context/Sequence length	Quantization	Deployment
Causal language models	deepseek-r1-0528	685B	62	128K	–	Official API
	qwen3-235b-a22b	235B	94	128K	–	Official API
	qwen3-32b	32.8B	64	32K	q4_K_M	Local, V100 16G × 4
	gemma-3-27b-it	27B	62	128K	q4_K_M	Local, V100 16G × 4
	gpt-oss-20b	21B	24	128K	MXFP4	Local, V100 16G × 4
Text embedding	qwen3-embedding-4b	4B	36	32K	–	Local, 4060Ti 16G × 2
Text reranking	qwen3-reranker-4b	4B	36	32K	–	Local, 4060Ti 16G × 2

Abbreviations: B, billion parameters; K, thousand; q4_K_M, 4-bit grouped quantization with per-channel scales and mixed precision; MXFP4, 4-bit mixed floating-point quantization; IT, instruction-tuned.

In the context of the RAG architecture, the present study proposes the development of a knowledge retrieval system, comprising a text embedding model and a re-ranking model. The text embedding model transforms radiological texts and relevant medical knowledge base content into high-dimensional vector representations, facilitating semantic retrieval. The re-ranking model subsequently optimizes the ordering of retrieved results to ensure greater contextual relevance to the input text. The knowledge base was constructed from a self-curated corpus, consisting of full-text radiological diagnostic guidelines for CAP and HAP. The documents were processed into text packages and indexed for retrieval during inference. In practical implementation, the RAG system integrates the retrieved external knowledge with the original radiology report before inputting it into the LLM, thereby enabling knowledge-enhanced diagnosis. This approach not only exploits the LLM’s strong capacity for processing unstructured language, but also enhances its ability to identify and interpret domain-specific medical information. The integration of LLMs and RAG can improve both diagnostic accuracy and interpretability, offering clinicians more credible and traceable diagnostic evidence. Detailed prompt design, inference workflow, and RAG configuration are provided under Text A in S1 Appendix.

### Task definition and model implementation

This study aims to automatically detect radiographic evidence of pneumonia from radiology text reports, formulating the task as a binary classification problem. The input consists of complete radiology reports, including findings, impressions, and conclusions, while the output is a binary label, with 0 representing the absence of radiographic evidence of pneumonia and 1 its presence. Each report is processed as input, and then integrated with relevant knowledge retrieved through the RAG architecture to support evidence identification. This streamlined and reproducible framework facilitates a systematic evaluation of various LLMs and their performance within the RAG setting, thereby establishing a standardized foundation for future performance benchmarking and clinical translation.

### Evaluation strategy

In order to systematically assess LLMs and their effectiveness within RAG frameworks, the present study has established a comprehensive, multidimensional evaluation system for detecting radiographic evidence of pneumonia based on radiological text reports. The evaluation metrics encompass accuracy, precision, sensitivity, specificity and F1-score. Accuracy is defined as the overall correctness of the model’s classifications; precision is defined as the proportion of correctly classified positive predictions; sensitivity is measured as the ability to correctly identify reports indicating radiographic evidence of pneumonia; specificity is evaluated as the capacity to correctly recognize reports without radiographic evidence of pneumonia; and the F1-score is captured as the balance between precision and recall, making it particularly suitable for imbalanced datasets. Point estimates were calculated from the full dataset, and 95% CIs were estimated using 1,000 patient-level clustered bootstrap replicates. All evaluated models were pre-trained large language models, and no additional training, fine-tuning, or dataset splitting was performed in this study. Model performance was assessed in an inference-only setting.

Initial model evaluation was performed on the MIMIC-IV Dataset, with expert annotations serving as the reference standard to ensure the reliability of the results. The same evaluation procedure was then applied to the GZ Dataset in order to assess the model’s generalization capability in an independent cohort. Utilizing a multi-metric evaluation framework, the study provides a comprehensive and objective assessment of various automated radiographic evidence detection from radiological texts, thereby offering a robust scientific basis for model selection and clinical implementation.

### Statistical analysis

All statistical analyses were conducted using R (version 4.5.0) and Python (version 3.12.9). Initially, the data were subjected to descriptive statistics in order to summarize the characteristics of the enrolled patients and their imaging reports. Continuous variables were reported as median (interquartile range, IQR) or mean ± standard deviation (SD), while categorical variables were expressed as frequency and percentage. The evaluation of model performance was conducted in strict accordance with the metrics and procedures delineated in the Evaluation Strategy section. As no model training was conducted, statistical analysis focused exclusively on performance evaluation metrics. In order to ensure the reliability of the results, all model outputs were cross-validated against expert-annotated empirical data. The execution of model inference, result extraction and performance computation was conducted through the utilization of automated scripts, with the objective of ensuring full reproducibility and consistency across the analytical process.

## Results

### Dataset characteristics

The present study comprised 1,799 patients from the MIMIC-IV Database, corresponding to a total of 29,560 imaging reports (see Table 2). Of these, 1,491 reports (from 69 patients) were chest CT examinations, and 28,069 reports (from 1,730 patients) were chest X-ray examinations. The median age of the study population was 64.0 [53.0, 75.0] years, with males accounting for 57.5%. The majority of patients (59.9%) were Caucasian and were primarily admitted from other types of ICUs (49.1%), medical ICUs (18.3%), and mixed medical-surgical ICUs (13.0%), with the remainder distributed among cardiac and cardiovascular ICUs. Admission years spanned from 2008 to 2019, exhibiting relatively balanced representation across respective periods (29.8% in 2008–2010, 21.7% in 2011–2013, 23.7% in 2014–2016, and 24.8% in 2017–2019). Furthermore, GZ Dataset’s baseline characteristics are provided in Table A in S1 Appendix.

**Table 2:** Baseline characteristics of the study population stratified by imaging modality in MIMIC IV Dataset.

Variables	Overall (patients, n = 1,799; reports, n = 29,560)	Chest CT (patients, n = 69; reports, n = 1,491)	CXR (patients, n = 1,730; reports, n = 28,069)
Age (years old)	64.00 [53.00, 75.00]	63.00 [53.00, 72.00]	64.00 [53.00, 75.00]
Sex (%)			
Male	1035 (57.5)	37 (53.6)	998 (57.7)
Female	764 (42.5)	32 (46.4)	732 (42.3)
Race (%)			
ASIAN	45 (2.5)	1 (1.4)	44 (2.5)
BLACK	153 (8.5)	5 (7.2)	148 (8.6)
HISPANIC/LATINO	64 (3.6)	0 (0.0)	64 (3.7)
OTHER	459 (25.5)	18 (26.1)	441 (25.5)
WHITE	1078 (59.9)	45 (65.2)	1033 (59.7)
Type of ICU (%)			
CCU	134 (7.4)	7 (10.1)	127 (7.3)
CVICU	220 (12.2)	9 (13.0)	211 (12.2)
MICU	329 (18.3)	16 (23.2)	313 (18.1)
MICU/SICU	233 (13.0)	11 (15.9)	222 (12.8)
OTHER ICU	883 (49.1)	26 (37.7)	857 (49.5)
Year of admission (%)			
2008 - 2010	536 (29.8)	16 (23.2)	520 (30.1)
2011 - 2013	390 (21.7)	13 (18.8)	377 (21.8)
2014 - 2016	427 (23.7)	16 (23.2)	411 (23.8)
2017 - 2019	446 (24.8)	24 (34.8)	422 (24.4)

Abbreviations: CT, computed tomography; CXR, Chest X-Ray; DR, digital radiography; ICU, intensive care unit; CCU, cardiac care unit; CVICU, cardiovascular intensive care unit; MICU, medical intensive care unit; MIMIC IV, medical information mart for intensive care IV; SICU, surgical intensive care unit.

### Model performance on different validation cohorts

Within the MIMIC-IV Dataset, LLMs and their implementations under RAG architectures exhibited significant variability in radiographic evidence detection performance for pneumonia. All results reported below were obtained from direct inference of pre-trained models without any dataset splitting or model training procedures. In the absence of retrieval augmentation, the gemma3-27b model demonstrated the most superior overall performance, attaining an accuracy of 0.892 (0.879, 0.916). The model demonstrated high specificity (0.966) alongside robust sensitivity (0.711) and an F1 score of 0.792. In comparison, qwen3-235b and deepseek-r1-0528 achieved accuracies of 0.856 and 0.812, respectively (Table 3). Following the integration of the RAG architecture, there was a marked improvement in the performance of the model, particularly in achieving a more balanced trade-off between sensitivity and specificity. Notably, the gemma3-27b model under the RAG framework demonstrated optimal performance (accuracy = 0.964, precision = 0.890, sensitivity = 0.999, F1 score = 0.941), reflecting excellent performance in identifying radiographic evidence of pneumonia. Subgroup analyses by imaging modality (CXR and CT) showed generally consistent performance trends (Table B in S1 Appendix). The overall model performance on the MIMIC-IV Dataset is summarized in Fig A in S1 Appendix.

**Table 3:** Predictive performance of candidate models in MIMIC IV Dataset.

Models	Accuracy (95%CI)	Precision (95%CI)	Sensitivity (95%CI)	Specificity (95%CI)	F1 score (95%CI)
deepseek-r1-0528	0.812 (0.791, 0.840)	0.630 (0.572, 0.672)	0.837 (0.792, 0.878)	0.801 (0.779, 0.837)	0.719 (0.673, 0.750)
qwen3-235b	0.856 (0.842, 0.885)	0.880 (0.833, 0.928)	0.582 (0.524, 0.641)	0.968 (0.958, 0.982)	0.700 (0.650, 0.749)
qwen3-32b	0.838 (0.824, 0.869)	0.925 (0.886, 0.969)	0.478 (0.419, 0.538)	0.984 (0.977, 0.995)	0.631 (0.575, 0.684)
gemma3-27b	0.892 (0.879, 0.916)	0.895 (0.854, 0.938)	0.711 (0.655, 0.764)	0.966 (0.957, 0.982)	0.792 (0.750, 0.831)
gpt-oss-20b	0.835 (0.820, 0.862)	0.915 (0.872, 0.957)	0.472 (0.410, 0.520)	0.982 (0.975, 0.992)	0.623 (0.564, 0.668)
rag-deepseek-r1-0528	0.815 (0.797, 0.844)	0.638 (0.587, 0.684)	0.826 (0.775, 0.867)	0.810 (0.795, 0.847)	0.720 (0.677, 0.755)
rag-qwen3-235b	0.897 (0.885, 0.922)	0.773 (0.733, 0.825)	0.908 (0.875, 0.944)	0.892 (0.878, 0.925)	0.835 (0.807, 0.870)
rag-qwen3-32b	0.906 (0.895, 0.930)	0.819 (0.780, 0.870)	0.866 (0.824, 0.903)	0.923 (0.912, 0.949)	0.842 (0.811, 0.877)
rag-gemma3-27b	0.964 (0.954, 0.977)	0.890 (0.856, 0.925)	0.999 (0.993, 1.000)	0.950 (0.938, 0.968)	0.941 (0.922, 0.960)
rag-gpt-oss-20b	0.857 (0.840, 0.883)	0.852 (0.805, 0.902)	0.611 (0.545, 0.657)	0.957 (0.947, 0.974)	0.712 (0.659, 0.750)

Abbreviations: CI, confidence interval; MIMIC IV, medical information mart for intensive care IV; RAG, retrieval-augmented generation.

In the GZ Dataset, while overall model performance demonstrated a modest decrease relative to MIMIC-IV Dataset, the performance trends remained stable. The non-augmented gemma3-27b model continued to demonstrate strong cross-domain generalization, while the gemma3-27b model under the RAG architecture maintained excellent classification performance (F1 = 0.926, sensitivity = 0.925). It is evident that other models, including deepseek-r1-0528 and qwen3-235b within the RAG framework, have also achieved high sensitivity and F1 scores (Table C in S1 Appendix). A visual comparison of the performance of the models is provided in Fig B in S1 Appendix.

### Pairwise comparison with the reference standard

In order to assess the performance of major LLMs within RAG architectures, a comparison was made between model predictions and reference standards that had been established by an expert panel. The confusion matrix analysis was employed to provide an intuitive evaluation of each model’s classification performance in detecting radiographic evidence of pneumonia, detailing the distribution of true positives (TP), false positives (FP), true negatives (TN), and false negatives (FN).

On the MIMIC-IV Dataset, all models performed satisfactorily in distinguishing presence versus absence of radiographic evidence of pneumonia, although their capabilities varied. Among the baseline LLMs, gemma3-27b and qwen3-235b achieved relatively higher classification accuracy, with substantially fewer FN compared to deepseek-r1-0528 and gpt-oss-20b (Fig C in S1 Appendix). Following the integration of the RAG architecture, a range of performance improvements were demonstrated by all models. It is notable that under the RAG framework, gemma3-27b delivered the most exceptional results. This is evidenced by substantial increases in both TP and TN, and only eight FN. This reflects a significantly enhanced ability to identify positive cases. The number of false positive results (1,053 cases) remained low, indicating a favorable balance between sensitivity and specificity. As demonstrated in the case of other RAG-enhanced models, such as qwen3-235b and qwen3-32b, reductions in FN were also exhibited, suggesting that retrieval-augmented mechanisms generally improve clinical consistency across models.

Across the GZ Dataset, the overall performance of the model exhibited comparable trends. In the case of the baseline LLMs, gemma3-27b continued to demonstrate relatively superior performance, whilst the integration of RAG architectures resulted in a further enhancement of classification accuracy (Fig D in S1 Appendix). The gemma3-27b model under the RAG framework achieved the best results, with 3,702 TP, 300 FN, 524 TN, and 296 FP. A marked improvement in both the ability to detect positive cases and the overall consistency of the test was observed in comparison with its unenhanced counterpart.

### Efficiency analysis

**Fig 3:**
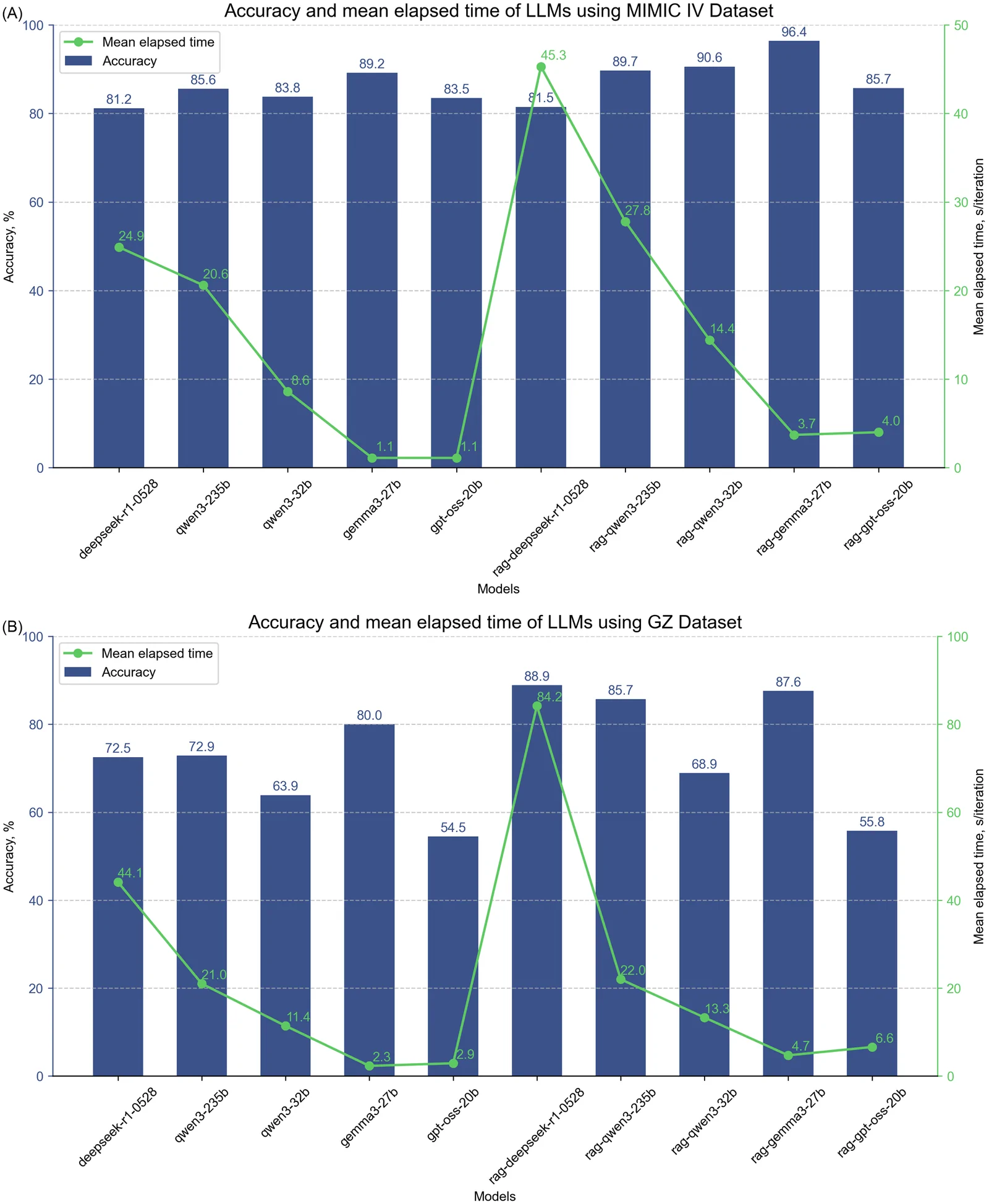
Comparison of accuracy and inference elapsed time across different models. Abbreviations: MIMIC IV, medical information mart for intensive care IV. Note: (A) accuracy and mean elapsed time for MIMIC IV Dataste. (B) accuracy and mean elapsed time for GZ Dataste The bar plot represents inference time per report for each model, and the line plot shows corresponding accuracy for pneumonia detection. Models are ordered along the x-axis for visual comparison.

In order to comprehensively assess the trade-off between detection accuracy and computational efficiency, the present study drew a comparison of major LLMs and their RAG architectures on the MIMIC-IV and GZ datasets, evaluating both accuracy and average inference time (Fig 3).

A significant disparity in computational efficiency was observed among models when the MIMIC-IV Dataset was analyzed. Among baseline LLMs, gemma3-27b exhibited remarkable efficiency, attaining an average inference time of merely 1.1 seconds while preserving high accuracy (89.2%). In contrast, deepseek-r1-0528 and the qwen3 series required 24.9 seconds and approximately 20 seconds per inference, respectively, reflecting considerably longer processing times. Following the integration of retrieval-augmented mechanisms, the majority of RAG models demonstrated a more favorable balance between accuracy and computational overhead. Within the RAG framework, gemma3-27b achieved the highest accuracy of 96.4%, while maintaining an average processing time of 3.7 seconds. In comparison, deepseek-r1-0528 required 45.3 seconds and qwen3-235b required 27.8 seconds to complete the same task. These results demonstrate that, under the RAG architecture, gemma3-27b combines superior classification performance with high computational efficiency, making it a strong candidate for efficient large-scale analysis of radiology text data rather than implying direct clinical performance advantages.

As evidenced by the GZ Dataset, the observed trends in model performance largely mirror those which were observed during the process of MIMIC-IV Dataset. Among baseline LLMs, gemma3-27b demonstrated the highest level of performance, achieving 80.0% accuracy with an average processing time of 2.3 seconds. In comparison, qwen3-32b and gpt-oss-20b exhibited faster computation but comparatively lower accuracy. The introduction of RAG architectures has been demonstrated to enhance overall model accuracy. Within the RAG-based models, gemma3-27b exhibited the optimal performance, attaining 87.6% accuracy with an average processing time of 4.7 seconds, thereby demonstrating robust cross-dataset stability. In contrast, deepseek-r1-0528, operating within the RAG framework, while achieving a high level of accuracy (88.9%), exhibited substantially elevated computation times (84.2 seconds). This discrepancy can be attributed to the divergent complexity of invoking the retrieval module across models.

## Discussion

The present study proposes and validates an LLM-driven automated radiographic evidence detection framework for pneumonia using radiology report texts from the MIMIC-IV database. Unlike traditional machine learning studies, this work does not involve model development or training, but instead focuses on benchmarking the performance of existing large language models in an inference-only setting. This addresses the existing gap in pneumonia diagnosis that lacks radiological evidence within critical care databases. Prior studies based on MIMIC have largely focused on risk prediction, pathogen analysis, and prognostic assessment, relying primarily on structured coding systems (e.g., ICD-9/10) with minimal integration of radiological text validation [23–25]. This conventional approach disregards the extensive clinical semantics inherent in imaging reports, despite the critical role of radiographic evidence in confirming pneumonia and supporting clinical decision-making. The integration of LLMs for semantic parsing and radiology report classification in this study has the potential to enhance the utilization of imaging information in database research, thereby establishing an innovative technical paradigm for text-based disease identification [26,27]. This methodological innovation represents a significant advancement, moving beyond traditional code-dependent analyses towards precise, semantically grounded radiographic evidence identification. It provides a new avenue for future clinical studies that integrate multi-source data. Importantly, this approach may have practical clinical implications by enabling efficient large-scale identification of pneumonia-related radiographic evidence from existing clinical text data, thereby supporting retrospective cohort construction and improving the scalability of clinical research workflows.

In recent years, there has been a notable increase in the utilization of LLMs in the field of medical big data analysis. The MIMIC database, which integrates both structured and unstructured information, has emerged as a pivotal platform for validation in this domain [28]. As demonstrated by extant research, LLMs have been shown to excel in a variety of tasks, including mental health assessment, prediction of adverse drug reactions, development of clinical practice guidelines, and forecasting disease-gene associations [29–32]. For instance, Liu et al. developed Meta General Practitioner (MetaGP) for clinical decision support, achieving remarkable performance [33]. Other studies have refined models such as BioBERT or ClinicalBERT using MIMIC data, enhancing their predictive accuracy and disease classification capabilities [34–36]. Despite these advances, the utilization of automated detection within radiology reports, particularly for the identification of pneumonia, remains significantly under-explored. Conventional natural language processing approaches are inadequate in their ability to capture the contextual dependencies and implicit diagnostic reasoning present in clinical reports. In contrast, LLMs offer advanced semantic comprehension and reasoning capabilities, thereby providing new opportunities for structured analysis of radiology texts. Against this backdrop, the present study investigates the potential of LLMs for classification-based recognition of radiographic evidence of pneumonia in radiology reports, addressing a key gap in current applications of large language models in medicine. From a clinical perspective, such automated text-based analysis may reduce the reliance on manual chart review and facilitate rapid screening of radiology reports, particularly in large databases where structured diagnostic coding is incomplete or delayed.

The findings suggest that models utilizing RAG demonstrate notable advantages in radiographic evidence classification for pneumonia. The RAG framework has been developed to enhance the model’s capacity to leverage specialized information and strengthen task robustness by dynamically retrieving relevant knowledge and integrating it with generative language models [37]. It is noteworthy that no clear positive correlation was identified between model performance and parameter scale. On the MIMIC-IV dataset, the smaller gemma3-27b model exhibited superior classification accuracy compared to the larger deepseek-r1-0528 model. This finding suggests that, for specific medical tasks, model suitability for the characteristics of the target task and radiology reports may be more critical than sheer parameter size. It is also possible that differences in language environment may have a significant impact on model performance. The pre-training data for most mainstream LLMs is predominantly in English, aligning closely with the linguistic characteristics of MIMIC’s English reports, while the structured format and precise terminology of these radiology reports facilitate the extraction of key diagnostic semantics. Furthermore, the time required for model inference increases with parameter scale. However, greater computational complexity does not necessarily result in proportional performance gains. This lends further support to the notion that ‘the optimal model is not the largest, but the most suitable.’ Although inference time provides a useful measure of computational efficiency, its direct impact on real-world clinical decision-making workflows requires further investigation in prospective settings. Therefore, the current findings should be interpreted as evidence of technical feasibility rather than direct clinical utility, and prospective validation in real-world clinical workflows remains necessary.

Despite the fact that this study presents a feasible and innovative research approach, it is important to acknowledge several limitations. Firstly, while utilizing a multicenter dataset, encompassing MIMIC-IV and radiology reports from a domestic tertiary hospital, enhances external validity and representativeness, heterogeneity may persist due to differences in linguistic structure, terminology, and radiological description styles across centers. Furthermore, the development cohort was restricted to patients with CCI, while the external validation cohort included general ICU patients. This population difference may affect model performance and limit generalizability. Secondly, the analysis is dependent on radiological text reports alone, excluding the incorporation of raw imaging data. Consequently, model inferences are indirectly constrained by the quality and completeness of report documentation. Thirdly, imaging performed ≥48 hours after ICU admission cannot fully distinguish hospital-acquired pneumonia from community-acquired pneumonia, and the study therefore focuses on detecting radiographic evidence of pneumonia rather than etiological classification, which may introduce potential misclassification. Moreover, the RAG architecture is contingent upon the quality and update frequency of external knowledge bases. It has been established that contemporary medical knowledge resources frequently demonstrate deficient structural organization and semantic coverage. This has the potential to impact the stability and accuracy of retrieval-augmented generation. This study primarily emphasizes model performance and computational efficiency, and the reference standard was established via expert consensus without formal inter-rater agreement metrics, which may limit reproducibility assessment. Only limited exploration of internal decision-making processes and interpretability was performed.

## Conclusions

This study demonstrates the feasibility of using LLMs for automated detection of radiographic evidence of pneumonia from radiology text reports in two datasets. The findings demonstrate that the RAG architecture leads to a substantial enhancement in both classification accuracy and generalization capability. It is important to note that model performance is not directly proportional to size: a medium-sized model (gemma3-27b) achieved optimal results, indicating that the alignment between model and task is more critical than the number of parameters. In summary, the performance of LMs in radiology text analysis is both efficient and robust, thus opening up new technical avenues for automated radiographic evidence detection in research settings, with potential for future clinical application pending further prospective validation.

## Supporting information

S1 Appendix Text A RAG Configuration and Implementation Details. **Table A** Baseline characteristics of the study population stratified by imaging modality in GZ Dataset. **Table B** Predictive performance of candidate models in MIMIC Dataset of Chest CT and Chest CXR. **Table C** Predictive performance of candidate models in GZ Dataset. **Fig A** Radar plot of LLM and RAG model performance in MIMIC IV Dataset. Abbreviations: F1, F1 score; RAG, retrieval-augmented generation; LLM, large language model, MIMIC IV, medical information mart for intensive care IV. Note: Each axis represents one evaluation metric: accuracy, precision, sensitivity, specificity, and F1 score. Metrics are calculated per report and normalized to the 0–1 range for visual comparison. The plot highlights differences in model performance, with rag-gemma3-27b showing the highest overall performance. **Fig B** Radar plot of LLM and RAG model performance in GZ Dataset Abbreviations: F1, F1 score; RAG, retrieval-augmented generation; LLM, large language model. Note: Each axis represents one evaluation metric: accuracy, precision, sensitivity, specificity, and F1 score. Metrics are calculated per report and normalized to the 0–1 range for visual comparison. The plot highlights differences in model performance, with rag-gemma3-27b showing the highest overall performance. **Fig C** Confusion matrices comparing reference standard with all models in MIMIC-IV Dataset. Note: Rows correspond to true labels (presence or absence of pneumonia), and columns correspond to predicted labels. Values are presented as counts of reports. **Fig D** Confusion matrices comparing reference standard with all models in GZ Dataset. Note: Rows correspond to true labels (presence or absence of pneumonia), and columns correspond to predicted labels. Values are presented as counts of reports.
(DOCX)
