# Supplementary material for: Large language models for pneumonia detection in radiology reports via text analysis
Source: PLOS Digit Health. 2026 Sep 23;5(9):e0001104. doi: 10.1371/journal.pdig.0001104 (PMC13600486; doi:10.1371/journal.pdig.0001104)
Supplement: Supplementary file 1 [file pdig.0001104.s001.docx]

**Text A: RAG Configuration and Implementation Details**

To facilitate reproducibility, the configuration and implementation details of the retrieval-augmented generation (RAG) framework are described below.

**Knowledge Base Construction**

The knowledge base consisted of domain-specific medical information on the radiographic features of pneumonia and other pulmonary conditions. Its content was curated to support the interpretation of radiology reports and improve the model’s reasoning during inference.

**Text Chunking Strategy**

The knowledge corpus was divided into fixed-length chunks, each containing a maximum chunk length of 1024 tokens. An overlap of 50 tokens was applied between adjacent segments to maintain contextual continuity.

**Embedding and Indexing**

The radiology reports and knowledge base chunks were encoded as dense vector representations using a text embedding model. The embeddings were indexed to enable efficient similarity-based retrieval.

**Retrieval Configuration**

For each input query, the three most relevant text chunks (k=3) were retrieved based on cosine similarity. The retrieved content was incorporated into the prompt context to guide model inference.

**Prompt Template**

The prompt template used in the RAG framework is provided below:

\# Role: Medical Imaging Diagnosis Specialist

\## Profile

- language: English

- description: Expert in analyzing medical imaging reports to detect signs of pneumonia

- background: Board-certified radiologist with 10+ years experience in pulmonary imaging

- personality: Precise, methodical, and conservative in diagnosis

- expertise: Interpretation of chest X-rays and CT scans for pulmonary conditions

- target_audience: Healthcare professionals seeking quick preliminary assessments

\## Skills

1. Diagnostic Interpretation

- Pneumonia detection: Identifying hallmark radiographic signs of pneumonia

- Differential diagnosis: Distinguishing pneumonia from other pulmonary conditions

- Report analysis: Extracting key findings from structured imaging reports

- Clinical correlation: Understanding limitations of imaging-only assessments

2. Decision Making

- The context inside <context></context> XML tags in an XML structure of the user input

- Please use the following context as your learned knowledge

- Read and comprehend the context information provided by the user

- Identify the question posed by the user based on the context information

- Utilize relevant details and knowledge from the context information to provide an accurate and concise answer to the user's question

- Ensure that the response is clear, direct, and relevant to the context

- Binary classification: Applying strict yes/no diagnostic criteria

- Uncertainty handling: Defaulting to conservative negative diagnosis

- Evidence evaluation: Requiring clear radiographic evidence for positive diagnosis

- Protocol adherence: Following standardized diagnostic algorithms

\## Rules

1. Diagnostic Principles:

- Only respond with "pneumonia" when report explicitly describes consolidation, air bronchograms, other definitive signs, pneumonia-related diagnosis, or phrases indicate pneumonia in the context

- Consider "no-pneumonia" as default response for ambiguous or incomplete reports

- Never speculate beyond the provided imaging findings

- Maintain strict binary classification without qualifications

2. Output Guidelines:

- Responses must be exactly either "pneumonia" or "no-pneumonia"

- No additional explanations, justifications, or commentary permitted

- English language responses only

- Case-sensitive lowercase output format

3. Limitations:

- Cannot request additional information or clarification beyond the content and context provided

- Must base assessment on provided report content and context

- No gradation of diagnostic certainty permitted

\## Workflows

- Goal: Provide binary classification of pneumonia presence from imaging reports

- Step 1: Scan report for definitive pneumonia indicators (consolidation, infiltrates, etc.)

- Step 2: If clear evidence found, respond "pneumonia"

- Step 3: For all other cases (ambiguous, negative, or insufficient data), respond "no-pneumonia"

- Expected Outcome: Single-word diagnostic classification

\## OutputFormat

1. Diagnostic Response:

- format: plain text

- structure: single word

- style: lowercase

- special_requirements: no punctuation or additional characters

2. Format Specifications:

- indentation: none

- sections: none

- highlighting: none

3. Validation Rules:

- validation: exact match to either "pneumonia" or "no-pneumonia"

- constraints: 11 characters maximum

- error_handling: system will reject any non-compliant output

4. Examples:

1) Example1:

- Title: Positive Diagnosis

- Format Type: text

- Note: Report clearly describes lobar consolidation

- Content: |

pneumonia

2) Example2:

- Title: Negative Diagnosis

- Format Type: text

- Note: Report shows clear lungs or insufficient data

- Content: |

no-pneumonia

**Decoding Parameters**

All models were configured to produce deterministic outputs. The temperature was set to 0, and the output length was constrained to a single token to match the binary classification task.

**Table A** Baseline characteristics of the study population stratified by imaging modality in GZ Dataset

| **Variables** | **Overall**  **(patients, n=1411; reports, n=4,822)** | **Chest CT**  **(patients, n=586; reports, n=1,882)** | **CXR**  **(patients, n=825; reports, n=2,940)** |
| --- | --- | --- | --- |
| Age (years old) | 68.00 [55.00, 79.00] | 67.00 [55.00, 78.00] | 70.00 [56.00, 80.00] |
| Sex (%) |  |  |  |
| Male | 921 (65.3) | 391 (66.7) | 530 (64.2) |
| Female | 490 (34.7) | 195 (33.3) | 295 (35.8) |

Abbreviations: CT, computed tomography; CXR, Chest X-Ray.

**Table B** Predictive performance of candidate models in MIMIC Dataset of Chest CT and Chest CXR

| **Imaging type** | **Models** | **Accuracy**  **(95%CI)** | **Precision**  **(95%CI)** | **Sensitivity**  **(95%CI)** | **Specificity**  **(95%CI)** | **F1 score**  **(95%CI)** |
| --- | --- | --- | --- | --- | --- | --- |
| Chest CT | deepseek-r1-0528 | 0.787 (0.751, 0.804) | 0.729 (0.696, 0.757) | 0.961 (0.946, 0.977) | 0.586 (0.510, 0.601) | 0.829 (0.805, 0.849) |
|  | qwen3-235b | 0.873 (0.847, 0.887) | 0.952 (0.928, 0.968) | 0.803 (0.771, 0.834) | 0.954 (0.927, 0.967) | 0.871 (0.848, 0.890) |
|  | qwen3-32b | 0.821 (0.793, 0.840) | 0.967 (0.946, 0.982) | 0.690 (0.653, 0.730) | 0.973 (0.952, 0.984) | 0.805 (0.777, 0.832) |
|  | gemma3-27b | 0.909 (0.887, 0.921) | 0.942 (0.921, 0.960) | 0.885 (0.856, 0.906) | 0.937 (0.909, 0.955) | 0.913 (0.893, 0.927) |
|  | gpt-oss-20b | 0.821 (0.788, 0.838) | 0.975 (0.949, 0.984) | 0.684 (0.647, 0.722) | 0.980 (0.955, 0.986) | 0.804 (0.773, 0.828) |
|  | rag-deepseek-r1-0528 | 0.826 (0.803, 0.849) | 0.814 (0.785, 0.847) | 0.877 (0.855, 0.909) | 0.767 (0.713, 0.794) | 0.844 (0.825, 0.869) |
|  | rag-qwen3-235b | 0.901 (0.876, 0.912) | 0.885 (0.854, 0.905) | 0.937 (0.917, 0.958) | 0.860 (0.808, 0.873) | 0.911 (0.889, 0.923) |
|  | rag-qwen3-32b | 0.892 (0.861, 0.901) | 0.921 (0.885, 0.933) | 0.874 (0.843, 0.898) | 0.913 (0.864, 0.921) | 0.897 (0.870, 0.908) |
|  | rag-gemma3-27b | 0.958 (0.942, 0.967) | 0.928 (0.902, 0.945) | 0.999 (0.998, 1.000) | 0.910 (0.872, 0.928) | 0.962 (0.949, 0.972) |
|  | rag-gpt-oss-20b | 0.843 (0.810, 0.858) | 0.961 (0.940, 0.978) | 0.737 (0.693, 0.766) | 0.965 (0.943, 0.979) | 0.834 (0.803, 0.855) |
| CXR | deepseek-r1-0528 | 0.813 (0.793, 0.840) | 0.621 (0.563, 0.666) | 0.824 (0.778, 0.869) | 0.809 (0.788, 0.841) | 0.708 (0.662, 0.746) |
|  | qwen3-235b | 0.856 (0.843, 0.884) | 0.871 (0.827, 0.925) | 0.558 (0.502, 0.620) | 0.968 (0.959, 0.984) | 0.680 (0.632, 0.736) |
|  | qwen3-32b | 0.839 (0.824, 0.867) | 0.918 (0.882, 0.969) | 0.457 (0.397, 0.511) | 0.985 (0.978, 0.995) | 0.610 (0.550, 0.660) |
|  | gemma3-27b | 0.892 (0.879, 0.916) | 0.889 (0.854, 0.936) | 0.693 (0.635, 0.749) | 0.967 (0.959, 0.982) | 0.779 (0.737, 0.821) |
|  | gpt-oss-20b | 0.836 (0.819, 0.865) | 0.906 (0.862, 0.956) | 0.450 (0.386, 0.508) | 0.982 (0.975, 0.992) | 0.601 (0.540, 0.657) |
|  | rag-deepseek-r1-0528 | 0.814 (0.796, 0.842) | 0.623 (0.566, 0.670) | 0.821 (0.770, 0.859) | 0.811 (0.792, 0.846) | 0.708 (0.661, 0.744) |
|  | rag-qwen3-235b | 0.896 (0.886, 0.923) | 0.763 (0.720, 0.814) | 0.906 (0.869, 0.943) | 0.893 (0.879, 0.923) | 0.828 (0.797, 0.865) |
|  | rag-qwen3-32b | 0.907 (0.895, 0.931) | 0.810 (0.771, 0.863) | 0.865 (0.820, 0.901) | 0.923 (0.913, 0.949) | 0.837 (0.808, 0.871) |
|  | rag-gemma3-27b | 0.964 (0.956, 0.978) | 0.886 (0.856, 0.926) | 0.999 (0.995, 1.000) | 0.951 (0.941, 0.970) | 0.939 (0.922, 0.961) |
|  | rag-gpt-oss-20b | 0.858 (0.840, 0.884) | 0.839 (0.786, 0.891) | 0.598 (0.530, 0.649) | 0.957 (0.945, 0.974) | 0.698 (0.643, 0.743) |

Abbreviations: CT, computed tomography; CXR, Chest X-Ray; CI, confidence interval; MIMIC IV, medical information mart for intensive care IV; RAG, retrieval-augmented generation.

**Table C** Predictive performance of candidate models in GZ Dataset

| **Models** | **Accuracy**  **(95%CI)** | **Precision**  **(95%CI)** | **Sensitivity**  **(95%CI)** | **Specificity**  **(95%CI)** | **F1 score**  **(95%CI)** |
| --- | --- | --- | --- | --- | --- |
| deepseek-r1-0528 | 0.725 (0.718, 0.773) | 0.973 (0.960, 0.986) | 0.688 (0.667, 0.730) | 0.906 (0.891, 0.960) | 0.806 (0.791, 0.835) |
| qwen3-235b | 0.729 (0.727, 0.783) | 0.970 (0.958, 0.984) | 0.695 (0.678, 0.745) | 0.896 (0.881, 0.955) | 0.810 (0.797, 0.842) |
| qwen3-32b | 0.639 (0.623, 0.681) | 0.980 (0.961, 0.990) | 0.576 (0.542, 0.611) | 0.943 (0.909, 0.976) | 0.726 (0.697, 0.751) |
| gemma3-27b | 0.800 (0.785, 0.834) | 0.959 (0.940, 0.970) | 0.794 (0.769, 0.825) | 0.834 (0.808, 0.902) | 0.868 (0.851, 0.887) |
| gpt-oss-20b | 0.545 (0.520, 0.583) | 0.977 (0.968, 0.995) | 0.463 (0.404, 0.476) | 0.948 (0.946, 0.991) | 0.628 (0.573, 0.641) |
| rag-deepseek-r1-0528 | 0.889 (0.875, 0.915) | 0.942 (0.920, 0.954) | 0.924 (0.913, 0.946) | 0.722 (0.704, 0.817) | 0.933 (0.920, 0.946) |
| rag-qwen3-235b | 0.857 (0.835, 0.879) | 0.950 (0.931, 0.963) | 0.873 (0.846, 0.893) | 0.776 (0.760, 0.868) | 0.910 (0.890, 0.921) |
| rag-qwen3-32b | 0.689 (0.686, 0.741) | 0.974 (0.960, 0.987) | 0.643 (0.626, 0.689) | 0.916 (0.897, 0.964) | 0.775 (0.761, 0.808) |
| rag-gemma3-27b | 0.876 (0.845, 0.888) | 0.926 (0.894, 0.932) | 0.925 (0.899, 0.937) | 0.639 (0.606, 0.731) | 0.926 (0.902, 0.930) |
| rag-gpt-oss-20b | 0.558 (0.545, 0.606) | 0.945 (0.920, 0.961) | 0.497 (0.461, 0.529) | 0.859 (0.840, 0.921) | 0.651 (0.617, 0.680) |

Abbreviations: CI, confidence interval; RAG, retrieval-augmented generation.

**Fig A** Radar plot of LLM and RAG model performance in MIMIC IV Dataset

Abbreviations: F1, F1 score; RAG, retrieval-augmented generation; LLM, large language model, MIMIC IV, medical information mart for intensive care IV.

Note: Each axis represents one evaluation metric: accuracy, precision, sensitivity, specificity, and F1 score. Metrics are calculated per report and normalized to the 0–1 range for visual comparison. The plot highlights differences in model performance, with rag-gemma3-27b showing the highest overall performance.

**Fig B** Radar plot of LLM and RAG model performance in GZ Dataset

Abbreviations: F1, F1 score; RAG, retrieval-augmented generation; LLM, large language model.

Note: Each axis represents one evaluation metric: accuracy, precision, sensitivity, specificity, and F1 score. Metrics are calculated per report and normalized to the 0–1 range for visual comparison. The plot highlights differences in model performance, with rag-gemma3-27b showing the highest overall performance.

**Fig C** Confusion matrices comparing reference standard with all models in MIMIC-IV Dataset

Note: Rows correspond to true labels (presence or absence of pneumonia), and columns correspond to predicted labels. Values are presented as counts of reports.

**Fig D** Confusion matrices comparing reference standard with all models in GZ Dataset

Note: Rows correspond to true labels (presence or absence of pneumonia), and columns correspond to predicted labels. Values are presented as counts of reports.
